# Obtaining information about cancer: prevalence and preferences among Japanese adults

**DOI:** 10.1186/s12889-015-1510-2

**Published:** 2015-02-14

**Authors:** Rina Miyawaki, Ai Shibata, Kaori Ishii, Koichiro Oka

**Affiliations:** Graduate School of Sport Sciences, Waseda University, Saitama, Japan; Faculty of Health and Sport Sciences, University of Tsukuba, Ibaraki, Japan; Faculty of Sport Sciences, Waseda University, Saitama, Japan

**Keywords:** Cancer information, Health communication, Cancer prevention, Japanese, Mass media

## Abstract

**Background:**

Providing information about cancer prevention might increase awareness of prevention and promote preventive behaviours. A better understanding about the prevalence and preferences of obtaining information about cancer might help to identify targeted individuals and design effective strategies for promoting cancer-preventive behaviours. Thus, the present study examined the prevalence and correlates of obtaining information about cancer among Japanese adults, and described preferences including source and content.

**Methods:**

Data were analysed for 3,058 Japanese adults (mean age 45.0 ± 13.4 years) who responded to an Internet-based cross-sectional survey. The data included whether information about cancer had been obtained, sources, preference for content, sociodemographic variables, health status, and cancer histories. Force-entry logistic regression analysis was used.

**Results:**

Overall, 46.7% of respondents had obtained information about cancer. Gender, age, and education level were statistically significant correlates of doing so. Women were more likely to obtain information (OR = 1.97) as were older age groups (40–49: OR = 1.54, 50–59: OR = 2.27, 60–69: OR = 3.83), those with higher education (2 years college or equivalent degree: OR = 1.31, college graduate or higher: OR = 1.48) and those with having cancer histories (personal: OR = 3.52, family: OR = 1.57, friends/co-worker: OR = 2.09). The most prevalent source of information about cancer was mass media. Content of prevention is most needed among inconsistent contents with the frequently obtained contents.

**Conclusions:**

Less than half of the respondents information about cancer. The finding suggests that better health communication strategies would be necessary to inform Japanese people about cancer. Understanding which subgroups were less likely to obtain information and preferences of information might be effective in promoting cancer prevention.

## Background

Cancer is the leading cause of death in Japan, and over 360,000 Japanese died of it in 2011 [1]. About half of all Japanese are expected to be diagnosed with cancer during their lifetime [1], and approximately 805,000 new cancer cases were diagnosed in 2010 [2]. However, cancer mortality can be reduced through screening and early detection [3,4]. Thus, it is important to enhance screening in the population. Moreover, cancer incidence would be preventable through lifestyle choice and behaviour changes such as stopping smoking, reducing alcohol consumption, eating a balanced diet, engaging in physical activity, and reducing BMI [1,5,6]. Indeed, the prior study estimated that 42.7% of Japanese cancer incidence and 42.6% of mortality could potentially be preventable [7]. However, general awareness of the attributable fraction of cancers being caused by major lifestyle factors was low in Japan [8].

Previous studies indicated that effective health communication could play a significant role in shaping individuals’ cancer-related decisions and behaviours [9-13]. Providing information about cancer prevention such as risk factors and preventive behaviours might increase awareness of prevention and promote such behaviours. Indeed, a number of researchers have indicated that obtaining information was positively associated with increased knowledge about cancer, and adoption of preventive and screening behaviours [14-17]. It is therefore necessary to develop effective health communication strategies that aim to inform about cancer based on solid evidence. A greater understanding about the prevalence and correlates of obtaining information about cancer might enable to identification of specific subgroup to target for interventions. Moreover, it might contribute to the design of effective strategies for promoting cancer-preventing behaviours. Several previous studies have identified that sociodemographic characteristics and cancer history were associated with cancer information seeking [9,14,18-21]. Recently, behaviours of obtaining information about cancer and the influence of obtaining cancer information on cancer preventive behaviours have been investigated by sociodemographic characteristics such as gender and race/ethnicity in the United States [10-13,22]. Nevertheless, little is known about how Japanese people obtain cancer information.

Moreover, to develop effective communication using cancer-related information, it is important to determine whether specific information sources are more effective than others and to evaluate needs for information about cancer. Hence, a number of studies about needs and sources of cancer information have been conducted with patients or their families [23]. However, few studies have assessed the preferences of the general public about sources and contents of cancer information.

Therefore, the present study examined the prevalence and demographic correlates of obtaining information about cancer among Japanese adults, and their preferences for sources and contents of cancer-related information.

## Methods

### Study design and participants

The current Internet-based cross-sectional study was conducted thorough a Japanese Internet research service company called MyVoice Communication, Inc. in November 2012. The company offers full-scale marketing research services, with approximately 300,000 voluntarily registered individuals, and detailed sociodemographic data for each. The sample size and parameters chosen were approximately 3,000 adult men and women aged between 20 and 69 years, with an equal number of men and women in each 10-year age bracket. Potential respondents (n = 9,767) were randomly and blindly selected from the registered samples and they were invited to participate in the Internet-based survey via e-mail. The number of potential respondents was determined by dividing the quota (n = 300) and by the response rate in each stratified sample group. This rate was estimated from the results of numerous previous surveys conducted by the Internet research service company. Internet-based questionnaires were placed in a protected area of a web site and potential respondents received a URL in an invitation e-mail. After more than 300 participants in each group had responded to the survey, acceptance of further participants was stopped in each group. A total of 3,292 respondents (response rate: 33.7%) voluntarily clicked on the “agree” button at the end of an online informed consent form and completed the questionnaire. Reward points valued at 70 yen were provided as incentives for participation (1 US dollar was equivalent to approximately 82 yen in 2012). The study was approved by the Ethics Committee of Waseda University, Japan.

### Measurements

#### Prevalence and demographic correlates of obtaining information about cancer

Participants were asked if they had obtained information about cancer from any source (had obtained/ had not obtained, yet). Possible sociodemographic correlates of obtaining information about cancer included gender, age (classified in years as 20–29, 30–39, 40–49, 50–59 and 60–69 years), marital status (currently married or not), education level (less than high school graduate, 2 years college or equivalent or college graduate), employment status (employed or not) and household income level (less than 5,000,000 yen, 5,000,000–10,000,000 yen and 10,000,000 yen or more). Health status was classified as good (very good and good) or poor (not good, poor, or don’t know). Personal, family and friend/co-worker cancer histories were categorized as having a history of cancer or not.

#### Preferences for sources and contents of cancer-related information

Participants were asked about the contents of cancer-related information both obtained and needed. The content options of cancer-related information were prevention, symptoms, screening/testing/early detection, diagnosis, treatment, prognosis or recovery, coping, medicine, medical expenses, cancer organization, alternative treatment, other and nothing. Respondents who only indicated that they had obtained information about cancer were asked about their information sources with multiple responses available (healthcare provider, family or friend, television or radio, Internet, newspaper, magazine, brochure or pamphlet, book, government health agency, other).

#### Statistical analysis

For the analysis, respondents with incomplete information were excluded (n = 234). Data were analysed for 3,058 people who provided complete information for the study variables. Chi-square tests were used to determine proportional differences between sociodemographic characteristics and whether or not the respondent had obtained information about cancer. A forced-entry logistic regression analysis was conducted to examine whether sociodemographic variables, perception of own health status and cancer history were related to obtaining information about cancer. Odds rations (ORs) and their 95% confidence intervals (CIs) were calculated. Statistical analyses were performed using SPSS for Windows version 22.0 J (Statistical Package for the Social Sciences; SPSS Inc., Chicago, IL, USA).

## Results

### Basic respondent characteristics

The respondent characteristics are summarized in Table 1. For the total sample, the mean age was 45.0 ± 13.4 years. Overall, 49.5% of samples were men, 64.4% were married, and 52.6% were employed. Of the respondents, 48.6% had graduated from college or graduate school, whereas 26.6% had less than a high school diploma. Twelve percent of the sample had a household income of more than 10,000,000 yen (10,000,000 yen was equivalent to approximately 122,000 US dollars) per year, whereas 46.2% earned less than 5,000,000 yen (5,000,000 yen was equivalent to approximately 61,000 US dollars) per year. Moreover, 4.1% had a personal history of cancer, 41.9% reported family history, and 45.2% reported a friend/co-worker history. More than three quarters, 76.1%, reported good health.

**Table 1:** **Sociodemographic characteristics, health status, cancer history of study participants (n = 3,058)**

	**Total**		**Obtained information about cancer**				
			**Yes (n = 1,458)**		**No (n = 1,600)**		**χ** ^**2**^ **p value**
	**n**	**%**	**n**	**%**	**n**	**%**	
**Gender**							
Men	1,513	49.5	590	40.5	923	57.7	***
Women	1,545	50.5	868	59.5	677	42.3	
**Age**							
20-29	544	17.8	158	10.8	386	24.1	***
30-39	632	20.7	224	15.4	408	25.5	
40-49	622	20.3	272	18.7	350	21.9	
50-59	629	20.6	363	24.9	266	16.6	
60-69	631	20.6	441	30.2	190	11.9	
**Marital status**							
Unmarried	1,090	35.6	411	28.2	679	42.4	***
Married	1,968	64.4	1,047	71.8	921	57.6	
**Education**							
Less than high school graduate	814	26.6	368	25.2	446	27.9	*
2 years college or equivalent	758	24.8	391	26.8	367	22.9	
College graduate	1,486	49.5	699	47.9	787	49.2	
**Employment status**							
Not employed	1,451	47.4	793	54.4	658	41.1	***
Employed	1,607	52.6	665	45.6	942	58.9	
**Household income**							
<5,000,000 yen	1,412	46.2	647	44.4	765	47.8	**
<10,000,00 yen	1,278	41.8	609	41.8	669	41.8	
≥10,000,000 yen	368	12.0	202	13.9	166	10.4	
**Health status**							
Poor	731	23.9	319	21.9	412	25.8	*
Good	2,327	76.1	1,139	78.1	1,188	74.3	
**Cancer history (Self)**							
No	2,934	95.9	1,354	92.9	1,580	98.8	***
Yes	124	4.1	104	7.1	20	1.3	
**Cancer history (Family)**							
No	1,776	58.1	699	47.9	1,077	67.3	***
Yes	1,282	41.9	759	52.1	523	32.7	
**Cancer history (Friend/co-worker)**							
No	1,682	55.0	587	40.3	1,095	68.4	***
Yes	1,383	45.2	878	60.2	505	31.6	

Abbreviations. *p < 0.05, **p < 0.01, ***p < 0.001.

### Prevalence and correlates of obtaining information about cancer

Overall, 46.7% (n = 1,458) of respondents had obtained information about cancer. Table 2 presents the results of the forced-entry regression analysis. Gender, age, and education level were the demographic factors with a statistically significant correlation to obtaining information about cancer. Women were more likely to obtain information about cancer than men (OR = 1.97, 95% CI = 1.64–2.39). Respondents aged 40–49 years (OR = 1.54, 95% CI = 1.17–2.03) or older (age 50–59 years: OR = 2.27, 95% CI = 1.71–3.02; age 60–69 years: OR = 3.83, 95% CI = 2.84–5.16) were positively associated with obtaining information about cancer. Those with 2 years college or equivalent degree (OR = 1.31, 95% CI = 1.05–1.64) and college graduates or higher (OR = 1.48, 95% CI = 1.21–1.81) were more likely to report obtaining information about cancer than the reference. In addition, respondents who perceived their health to be good (OR = 1.23, 95% CI = 1.02–1.48) were also more likely to obtain information about cancer than those who perceived their health to be poor. Previous experience of cancer in self, family or friend was positively associated with obtaining information about cancer (respondents with cancer diagnosis: OR = 3.52, 95% CI = 2.12–5.85; having family cancer history: OR = 1.57, 95% CI = 1.34–1.85; having friends/co-worker with cancer history: OR = 2.09, 95% CI = 1.77–2.47).

**Table 2:** **Multivariate logistic models for obtaining information about cancer**

	**OR**	**(95% CI)**	**p value**	**AOR**	**(95% CI)**	**p value**
**Gender**						
Women	2.01	(1.72.32)	***	1.97	(1.64-2.39)	***
**Age**						
20-29	1	(ref)		1	(ref)	
30-39	1.34	(1.05-1.72)	*	1.24	(0.95-1.63)	
40-49	1.90	(1.49-2.42)	***	1.54	(1.17-2.03)	**
50-59	3.33	(2.61-4.25)	***	2.27	(1.71-3.02)	***
60-69	5.67	(4.41-7.29)	***	3.83	(2.84-5.16)	***
**Marital status**						
Married	1.88	(1.62-2.19)	***	1.04	(0.86-1.25)	
**Education**						
Less than high school graduate	1	(ref)		1	(ref)	
2 years college or equivalent	1.29	(1.06-1.57)		1.31	(1.05-1.64)	*
College graduate	1.08	(0.91-1.28)	*	1.48	(1.21-1.81)	***
**Employment status**						
Employed	0.59	(0.51-0.68)	***	0.88	(0.73-1.07)	
**Household income**						
<5,000,000 yen	1	(ref)		1	(ref)	
<10,000,00 yen	1.08	(0.93-1.25)	**	0.99	(0.84-1.19)	
≥10,000,000 yen	1.44	(1.14-1.81)		1.24	(0.95-1.61)	
**Health status**						
Good	1.24	(1.05-1.46)	*	1.23	(1.02-1.48)	*
**Cancer History (Self)**						
Yes	6.07	(3.74-9.85)	***	3.52	(2.12-5.85)	***
**Cancer History (Family)**						
Yes	2.24	(1.93-2.59)	***	1.57	(1.34-1.85)	***
**Cancer History (Friend/co-worker)**						
Yes	3.28	(2.83-3.81)	***	2.09	(1.77-2.47)	***

*Abbreviations.* *OR* odds ratio, *CI* confidence interval, *ref* referent group.

*p < 0.05, **p < 0.01, ***p < 0.001.

### Cancer information preferences

Table 3 shows preferred sources of information about cancer. The most prevalent cancer information sources among Japanese people were mass media. The most reported source was television/radio (68.4%) followed by the Internet (63.7%), and newspaper (43.3%). Approximately 40% of respondents obtained information from either family or friend (41.0%) or health care provider (38.5%).

**Table 3:** **Source of information for participants who had obtained information about cancer (n = 1,458)**

	**Source of cancer information**	
**Information source**	**n**	**%**
Health care provider	561	38.5
Family, Friend	598	41.0
Television, Radio	997	68.4
Internet	929	63.7
Newspaper	631	43.3
Magazine	311	21.3
Broshure, Pamphlet	225	15.4
Book	227	15.6
Government health agency	243	16.7
Other	18	1.2

Respondents are not mutually exclusive.

Table 4 shows the content of the cancer-related information that participants obtained and expected to obtain. Of the total, 16.9% of respondents obtained information about screening or testing, 13.5% about symptoms, and 12.8% about treatment. The other side, the most frequently wanted content of the cancer-related information was screening or testing (43.7%) followed by symptoms (34.5%) as well as obtained content. Around one third, 34% of respondents needed the information about prevention. The percentage of respondents who expected to obtain information about prevention was higher than the percentage that actually obtained it.

**Table 4:** **Content of cancer-related information that participants obtained and needed (n = 3,058)**

	**Obtained information**		**Needed information**	
**Contents of information**	**n**	**%**	**n**	**%**
Screening/Testing/Early detection	516	16.9	1,336	43.7
Symptoms	414	13.5	1,056	34.5
Treatment cure	391	12.8	1,019	33.3
Prevention	299	9.8	1,041	34.0
Diagnosis	272	8.9	766	25.0
Medical expenses	206	6.7	630	20.6
Medicine	193	6.3	630	20.6
Cancer organization	130	4.3	517	16.9
Prognosis/recovery	104	3.4	479	15.7
Coping	84	2.7	546	17.9
Alternative treatment	69	2.3	328	10.7
Other	2	0.1	10	0.3

Respondents are not mutually exclusive.

## Discussion

This is the first study assessing behaviours and preferences around obtaining information about cancer in Japan. Information about cancer had been obtained by less than half of those responding, which was similar to levels seen in previous studies [18,20,21,24]. However, this level may not be sufficient. This result implies that efforts to inform people about cancer might be not enough to reach a large proportion of the population. Effective health communication strategies about cancer are therefore required to promote cancer preventing lifestyle changes, including attending cancer screening.

The current study found that some sociodemographic characteristics are significantly correlated with obtaining information about cancer. Women were more likely to obtain information about cancer, which is consistent with previous studies [6,18,21]. In contrast, the number of deaths from and incidence of cancer was larger in men. The results suggest that a communication strategy focused on men is particularly important. Older age groups were found to be more likely to obtain information about cancer among Japanese adults. This result is inconsistent with prior studies that indicated older respondents were less likely to seek information about cancer [18,20,21,25]. People often tend to seek and receive cancer information from mass media including television and print media [26]. In Japan, older people tend to watch more television [27] and have a higher rate of newspaper subscriptions [28] than other age groups. Thus, older age groups may also have more opportunity to obtain information about cancer. The finding suggests that the information about cancer is not reaching younger people compared to older people. It is important for a young age to adopt preventive behaviours before getting cancer. Furthermore, cancer has been increasing across all age groups, including younger people recently [1]. Thus, greater effort to inform younger people is needed. Respondents with a previous history of cancer or those with family, friends, or co-workers having a history of cancer were more likely to obtain information about cancer. Experience of feeling closer to cancer might enhance their interest in cancer compared with those not having cancer history.

This study also examined the preferred sources of information about cancer. Mass media such as television/radio, Internet, and newspaper were the most common source of information about cancer among Japanese adults. These findings were consistent with those of other studies [12,18,21]. Previous study indicated that mass media had a considerable influence on cancer communications, because it could attract and hold the attention of those at cancer risk [29]. Moreover, attracting and holding public attention through mass media could help motivate individual behavioural change [26]. The effective use of mass media could contribute to delivering cancer information to Japanese adults. Therefore, future research should explore specific and effective ways to using mass media.

Additionally, the present study revealed the contents of cancer information both obtained and needed. The most frequently obtained content was about screening, symptoms, and treatment, which is consist with a previous survey [18,30]. In the present study, contents of cancer information which respondents needed were similar to contents which respondents obtained. However, some needed contents of cancer information were inconsistent with the frequently obtained content such as prevention, medical expenses, and coping. The finding suggests that it is necessary to provide additional information to satisfy expectations. In particular, contents of prevention are needed to inform because it was the most needed information among inconsistent contents of information. This might also increase awareness and promote behavioural change to support cancer prevention. Mass media, which was the main cancer information source, might be able to provide information about cancer prevention effectively. However, previous studies indicated that mass media tend to focus on cancer treatment and patients rather than on prevention [31-33]. Furthermore, the distinctive characteristics of Japanese mass media coverage are not well understood. Thus, additional research on how cancer information is presented by Japanese mass media is needed.

The current study had some limitations. First, this study was conducted in an Internet setting. Selection bias is a major factor known to limit the generalizability of results in the Internet survey. It occurs because of the non-representative nature of the Internet population and the self-selection of participants [34,35]. However, this study has random selection of participants, an equally distributed gender (men or women) and age group (20–69 years) from a pool of 300,000 people. These may have helped to mitigate bias and ensure greater representativeness in the survey. Second, data are susceptible to responder bias because of self-reported data. Third, the present study used a self-administered questionnaire to examine obtaining information about cancer. Although the questionnaire was adopted from the previous survey, psychometric properties of scales had not evaluated. Therefore, the reliability and validity of the scales were insufficient. Finally, present study was the lack of clarity between active and passive information obtaining behaviours. Furthermore, lack of differentiating between obtaining information for one-self and obtaining information for others was another limitation.

## Conclusions

The prevalence of individuals obtaining information about cancer among Japanese adults through internet-based study was not sufficient. The result highlights a need for effective health communication strategies to inform people about cancer. In particular, communication efforts should be targeted towards men and younger people. Additionally, mass media was the most common source of information about cancer, and information was required about prevention as well as screening, symptoms and treatment. The present study would contribute to the development of health communication strategies to providing Japanese people with information about cancer by understanding subgroup with low obtaining information and preferences including information source and content.
